# Supplementary material for: Hot spot prediction in protein-protein interactions by an ensemble system
Source: BMC Syst Biol. 2018 Dec 31;12(Suppl 9):132. doi: 10.1186/s12918-018-0665-8 (PMC6311905; doi:10.1186/s12918-018-0665-8)
Supplement: Supplementary file 2 — All relevant MATLAB code. A simple Matlab implement of our predictor. (ZIP 19,742 kb) [file 12918_2018_665_MOESM2_ESM.zip › Additional file 2/Read ASA/ASA website/dbMPIKT/3BN9A.html]

 NetSurfP - Protein Surface Accessibility and Secondary Structure Predictions

  

|  |  |  |
| --- | --- | --- |
|  |  | NetSurfP - Protein Surface Accessibility and Secondary Structure Predictions  Technical University of Denmark |

  


---

```
# For publication of results, please cite:
# A generic method for assignment of reliability scores applied to solvent accessibility predictions.
# Bent Petersen, Thomas Nordahl Petersen, Pernille Andersen, Morten Nielsen and Claus Lundegaard
# BMC Structural Biology 2009, 9:51 doi:10.1186/1472-6807-9-51
#
# Column 1: Class assignment - B for buried or E for Exposed - Threshold: 25% exposure, but not based on RSA
# Column 2: Amino acid
# Column 3: Sequence name
# Column 4: Amino acid number
# Column 5: Relative Surface Accessibility - RSA
# Column 6: Absolute Surface Accessibility
# Column 7: Z-fit score for RSA prediction
# Column 8: Probability for Alpha-Helix
# Column 9: Probability for Beta-strand
# Column 10: Probability for Coil
E V  3BN9A                  1    0.308  47.293  -2.262   0.005   0.045   0.951
B V  3BN9A                  2    0.151  23.239  -2.502   0.004   0.514   0.481
E G  3BN9A                  3    0.367  28.914  -1.121   0.004   0.420   0.576
B G  3BN9A                  4    0.259  20.391  -1.453   0.020   0.205   0.775
E T  3BN9A                  5    0.543  75.314   1.110   0.021   0.279   0.699
E D  3BN9A                  6    0.594  85.567   1.199   0.020   0.205   0.775
B A  3BN9A                  7    0.055   6.072  -0.059   0.018   0.088   0.893
E D  3BN9A                  8    0.552  79.558   1.298   0.018   0.047   0.935
E E  3BN9A                  9    0.497  86.808   0.129   0.018   0.047   0.935
E G  3BN9A                 10    0.513  40.389  -0.395   0.018   0.019   0.964
E E  3BN9A                 11    0.507  88.520   0.956   0.018   0.019   0.964
B W  3BN9A                 12    0.153  36.821   1.299   0.004   0.138   0.858
B P  3BN9A                 13    0.102  14.545   0.851   0.005   0.336   0.660
B W  3BN9A                 14    0.057  13.757   0.689   0.018   0.846   0.136
B Q  3BN9A                 15    0.053   9.519   0.659   0.006   0.962   0.032
B V  3BN9A                 16    0.019   2.905   1.096   0.006   0.962   0.032
B S  3BN9A                 17    0.029   3.364   0.233   0.001   0.959   0.040
B L  3BN9A                 18    0.018   3.204   1.360   0.001   0.959   0.040
B H  3BN9A                 19    0.106  19.190   0.699   0.001   0.959   0.040
B A  3BN9A                 20    0.164  18.018  -0.078   0.002   0.816   0.182
E L  3BN9A                 21    0.437  80.088   0.136   0.005   0.262   0.733
E G  3BN9A                 22    0.689  54.209  -2.181   0.005   0.015   0.979
E Q  3BN9A                 23    0.566 101.141  -1.353   0.005   0.015   0.979
E G  3BN9A                 24    0.371  29.198   0.615   0.004   0.138   0.858
B H  3BN9A                 25    0.096  17.371  -1.040   0.004   0.420   0.576
B I  3BN9A                 26    0.104  19.203   0.450   0.003   0.718   0.279
B C  3BN9A                 27    0.014   1.980   1.101   0.003   0.718   0.279
B G  3BN9A                 28    0.043   3.384  -1.093   0.023   0.655   0.322
B A  3BN9A                 29    0.033   3.615  -0.899   0.021   0.756   0.223
B S  3BN9A                 30    0.052   6.059   0.132   0.018   0.846   0.136
B L  3BN9A                 31    0.050   9.137   0.215   0.021   0.756   0.223
B I  3BN9A                 32    0.059  10.823   0.474   0.021   0.451   0.528
B S  3BN9A                 33    0.136  15.881   0.653   0.019   0.141   0.840
E P  3BN9A                 34    0.656  93.115   0.603   0.018   0.019   0.964
E N  3BN9A                 35    0.316  46.218   0.957   0.018   0.047   0.935
B W  3BN9A                 36    0.064  15.440   1.323   0.023   0.655   0.322
B L  3BN9A                 37    0.021   3.900   1.275   0.018   0.846   0.136
B V  3BN9A                 38    0.023   3.566   1.556   0.011   0.918   0.071
B S  3BN9A                 39    0.030   3.504   0.562   0.011   0.918   0.071
B A  3BN9A                 40    0.022   2.402   0.777   0.021   0.756   0.223
B A  3BN9A                 41    0.029   3.240  -0.461   0.079   0.592   0.329
B H  3BN9A                 42    0.060  10.987   0.715   0.079   0.592   0.329
B C  3BN9A                 43    0.015   2.036   1.495   0.074   0.484   0.442
B Y  3BN9A                 44    0.046   9.894   0.527   0.066   0.296   0.638
B I  3BN9A                 45    0.280  51.856   0.632   0.056   0.142   0.802
E D  3BN9A                 46    0.363  52.279  -0.048   0.053   0.043   0.903
E D  3BN9A                 47    0.432  62.280  -0.758   0.058   0.017   0.925
E R  3BN9A                 48    0.528 120.958  -0.980   0.053   0.043   0.903
E G  3BN9A                 49    0.486  38.240  -0.761   0.113   0.043   0.844
E F  3BN9A                 50    0.446  89.492  -1.250   0.053   0.043   0.903
E R  3BN9A                 51    0.484 110.813  -1.082   0.018   0.088   0.893
E Y  3BN9A                 52    0.367  78.471  -1.362   0.018   0.088   0.893
E S  3BN9A                 53    0.338  39.637  -1.183   0.018   0.088   0.893
E D  3BN9A                 54    0.441  63.548   0.086   0.005   0.045   0.951
B P  3BN9A                 55    0.165  23.456  -1.180   0.004   0.085   0.910
E T  3BN9A                 56    0.370  51.361  -0.746   0.005   0.262   0.733
E Q  3BN9A                 57    0.295  52.705   1.169   0.004   0.616   0.381
B W  3BN9A                 58    0.033   7.961   0.554   0.001   0.900   0.099
B T  3BN9A                 59    0.146  20.181   1.669   0.001   0.959   0.040
B A  3BN9A                 60    0.015   1.675   1.422   0.000   0.983   0.017
B F  3BN9A                 61    0.077  15.374   1.113   0.001   0.959   0.040
B L  3BN9A                 62    0.023   4.230   0.861   0.001   0.959   0.040
B G  3BN9A                 63    0.042   3.290  -0.636   0.002   0.816   0.182
B L  3BN9A                 64    0.169  30.944  -0.256   0.023   0.655   0.322
B H  3BN9A                 65    0.140  25.448   0.608   0.004   0.616   0.381
B D  3BN9A                 66    0.281  40.550  -0.200   0.022   0.552   0.426
B Q  3BN9A                 67    0.145  25.951  -0.107   0.021   0.279   0.699
E S  3BN9A                 68    0.419  49.083  -1.057   0.020   0.205   0.775
E Q  3BN9A                 69    0.515  91.925   0.201   0.020   0.205   0.775
E R  3BN9A                 70    0.512 117.179  -0.580   0.018   0.088   0.893
E S  3BN9A                 71    0.662  77.575  -1.316   0.018   0.088   0.893
E A  3BN9A                 72    0.573  63.123  -1.889   0.018   0.047   0.935
E P  3BN9A                 73    0.565  80.230  -0.458   0.018   0.047   0.935
E G  3BN9A                 74    0.492  38.744  -0.412   0.004   0.197   0.799
E V  3BN9A                 75    0.344  52.857  -0.645   0.004   0.616   0.381
E Q  3BN9A                 76    0.246  44.007   0.829   0.001   0.900   0.099
E E  3BN9A                 77    0.451  78.720   0.839   0.001   0.959   0.040
B R  3BN9A                 78    0.102  23.427   1.845   0.001   0.959   0.040
E R  3BN9A                 79    0.420  96.157   1.410   0.001   0.900   0.099
B L  3BN9A                 80    0.033   6.061  -0.203   0.002   0.816   0.182
E K  3BN9A                 81    0.447  91.907   1.229   0.001   0.900   0.099
E R  3BN9A                 82    0.309  70.692   1.267   0.001   0.959   0.040
B I  3BN9A                 83    0.060  11.045   0.658   0.001   0.959   0.040
B I  3BN9A                 84    0.116  21.460   0.726   0.001   0.959   0.040
B S  3BN9A                 85    0.193  22.584   0.573   0.002   0.816   0.182
B H  3BN9A                 86    0.063  11.532   0.846   0.004   0.197   0.799
E P  3BN9A                 87    0.488  69.261   0.216   0.005   0.045   0.951
E F  3BN9A                 88    0.504 101.073  -0.113   0.005   0.015   0.979
B F  3BN9A                 89    0.097  19.428  -0.114   0.005   0.045   0.951
E N  3BN9A                 90    0.370  54.139   0.547   0.018   0.047   0.935
E D  3BN9A                 91    0.535  77.021  -0.696   0.018   0.019   0.964
E F  3BN9A                 92    0.657 131.800  -1.562   0.018   0.019   0.964
E T  3BN9A                 93    0.462  64.135  -0.201   0.018   0.019   0.964
B F  3BN9A                 94    0.212  42.548   0.406   0.018   0.047   0.935
E D  3BN9A                 95    0.479  69.038  -0.275   0.018   0.047   0.935
B Y  3BN9A                 96    0.102  21.733   0.548   0.018   0.088   0.893
B D  3BN9A                 97    0.040   5.764  -0.434   0.022   0.359   0.619
B I  3BN9A                 98    0.027   5.051   1.016   0.018   0.846   0.136
B A  3BN9A                 99    0.018   2.006   1.253   0.011   0.918   0.071
B L  3BN9A                100    0.019   3.534   1.481   0.011   0.918   0.071
B L  3BN9A                101    0.028   5.108   1.015   0.006   0.962   0.032
B E  3BN9A                102    0.204  35.691   1.764   0.001   0.900   0.099
B L  3BN9A                103    0.034   6.207  -0.088   0.002   0.816   0.182
E E  3BN9A                104    0.461  80.484   1.162   0.022   0.359   0.619
E K  3BN9A                105    0.502 103.261   0.733   0.021   0.279   0.699
E P  3BN9A                106    0.412  58.505   0.344   0.021   0.279   0.699
B A  3BN9A                107    0.063   6.954   0.083   0.022   0.359   0.619
E E  3BN9A                108    0.588 102.724   1.393   0.022   0.359   0.619
E Y  3BN9A                109    0.276  59.045   0.053   0.020   0.205   0.775
E S  3BN9A                110    0.363  42.508  -0.522   0.018   0.047   0.935
E S  3BN9A                111    0.685  80.270  -0.097   0.005   0.045   0.951
E M  3BN9A                112    0.328  65.713   0.644   0.005   0.336   0.660
B V  3BN9A                113    0.051   7.777   0.438   0.003   0.718   0.279
B R  3BN9A                114    0.251  57.365   1.200   0.002   0.816   0.182
B P  3BN9A                115    0.095  13.438   0.326   0.018   0.846   0.136
B I  3BN9A                116    0.025   4.625   0.904   0.018   0.846   0.136
B S  3BN9A                117    0.093  10.946   0.006   0.003   0.718   0.279
B L  3BN9A                118    0.038   6.903   0.801   0.005   0.262   0.733
B P  3BN9A                119    0.084  11.905   0.142   0.018   0.019   0.964
E D  3BN9A                120    0.466  67.208   0.973   0.018   0.019   0.964
E A  3BN9A                121    0.485  53.480  -0.480   0.018   0.019   0.964
E S  3BN9A                122    0.719  84.279  -1.903   0.018   0.019   0.964
E H  3BN9A                123    0.334  60.846  -0.134   0.018   0.047   0.935
E V  3BN9A                124    0.589  90.575   0.374   0.018   0.088   0.893
B F  3BN9A                125    0.077  15.414   0.109   0.018   0.088   0.893
E P  3BN9A                126    0.529  75.008   0.475   0.018   0.019   0.964
E A  3BN9A                127    0.637  70.142  -0.087   0.018   0.019   0.964
E G  3BN9A                128    0.543  42.710  -0.996   0.005   0.015   0.979
B K  3BN9A                129    0.240  49.306   0.287   0.005   0.045   0.951
E A  3BN9A                130    0.325  35.826   1.682   0.004   0.616   0.381
B I  3BN9A                131    0.015   2.830   1.428   0.001   0.900   0.099
B W  3BN9A                132    0.092  22.150   1.377   0.001   0.959   0.040
B V  3BN9A                133    0.017   2.613   1.046   0.001   0.959   0.040
B T  3BN9A                134    0.053   7.351   0.378   0.001   0.900   0.099
B G  3BN9A                135    0.036   2.794  -1.592   0.003   0.718   0.279
B W  3BN9A                136    0.115  27.658   0.412   0.021   0.451   0.528
B G  3BN9A                137    0.062   4.895  -1.772   0.022   0.359   0.619
E H  3BN9A                138    0.317  57.753   0.499   0.021   0.451   0.528
B T  3BN9A                139    0.174  24.120  -0.018   0.021   0.279   0.699
E Q  3BN9A                140    0.455  81.227   0.377   0.019   0.141   0.840
E Y  3BN9A                141    0.671 143.478  -0.480   0.018   0.047   0.935
E G  3BN9A                142    0.711  55.948  -1.702   0.005   0.015   0.979
E G  3BN9A                143    0.412  32.464  -1.375   0.005   0.015   0.979
E T  3BN9A                144    0.645  89.503  -0.899   0.018   0.019   0.964
E G  3BN9A                145    0.355  27.907  -0.159   0.058   0.017   0.925
B A  3BN9A                146    0.148  16.299  -0.744   0.181   0.016   0.803
E L  3BN9A                147    0.559 102.371   0.246   0.354   0.048   0.598
E I  3BN9A                148    0.253  46.786   1.669   0.307   0.165   0.527
B L  3BN9A                149    0.045   8.313   0.185   0.231   0.330   0.439
B Q  3BN9A                150    0.241  43.132   0.978   0.169   0.612   0.219
E K  3BN9A                151    0.350  71.892   1.011   0.072   0.855   0.073
B G  3BN9A                152    0.030   2.345   0.468   0.011   0.918   0.071
E E  3BN9A                153    0.466  81.480   0.579   0.018   0.846   0.136
B I  3BN9A                154    0.044   8.048   0.765   0.021   0.756   0.223
E R  3BN9A                155    0.348  79.738   0.869   0.023   0.655   0.322
B V  3BN9A                156    0.149  22.932   0.236   0.023   0.655   0.322
B I  3BN9A                157    0.161  29.748   1.039   0.004   0.616   0.381
E N  3BN9A                158    0.421  61.590   1.085   0.021   0.279   0.699
E Q  3BN9A                159    0.253  45.186   0.655   0.694   0.003   0.303
E T  3BN9A                160    0.712  98.685   1.407   0.858   0.002   0.139
E T  3BN9A                161    0.403  55.952   2.259   0.879   0.010   0.111
B C  3BN9A                162    0.015   2.078   1.203   0.879   0.010   0.111
E E  3BN9A                163    0.361  63.102   1.262   0.879   0.010   0.111
E N  3BN9A                164    0.668  97.751   1.114   0.802   0.014   0.185
E L  3BN9A                165    0.293  53.648   1.046   0.802   0.014   0.185
B L  3BN9A                166    0.137  25.066   0.354   0.600   0.003   0.397
E P  3BN9A                167    0.633  89.794   0.174   0.246   0.004   0.750
E Q  3BN9A                168    0.634 113.161  -0.661   0.181   0.016   0.803
E Q  3BN9A                169    0.463  82.781   1.372   0.113   0.043   0.844
B I  3BN9A                170    0.051   9.398   0.066   0.052   0.084   0.864
E T  3BN9A                171    0.266  36.936   1.204   0.018   0.019   0.964
E P  3BN9A                172    0.638  90.490   0.221   0.018   0.019   0.964
E R  3BN9A                173    0.348  79.646   0.342   0.018   0.019   0.964
B M  3BN9A                174    0.037   7.384   0.026   0.020   0.205   0.775
B M  3BN9A                175    0.038   7.664   0.265   0.022   0.552   0.426
B C  3BN9A                176    0.017   2.331   0.898   0.023   0.655   0.322
B V  3BN9A                177    0.029   4.380   0.015   0.021   0.451   0.528
B G  3BN9A                178    0.101   7.917  -0.635   0.020   0.205   0.775
B F  3BN9A                179    0.224  44.937   0.696   0.019   0.141   0.840
E L  3BN9A                180    0.590 108.029   0.188   0.018   0.047   0.935
E S  3BN9A                181    0.764  89.553   0.023   0.018   0.019   0.964
E G  3BN9A                182    0.486  38.264  -1.233   0.018   0.019   0.964
E G  3BN9A                183    0.447  35.210  -0.883   0.018   0.047   0.935
E V  3BN9A                184    0.431  66.275   0.565   0.019   0.141   0.840
B D  3BN9A                185    0.115  16.543  -2.272   0.020   0.205   0.775
B S  3BN9A                186    0.133  15.599   0.023   0.020   0.205   0.775
B C  3BN9A                187    0.035   4.858  -0.764   0.021   0.279   0.699
E Q  3BN9A                188    0.354  63.224   0.151   0.020   0.205   0.775
B G  3BN9A                189    0.227  17.849  -1.274   0.018   0.088   0.893
B D  3BN9A                190    0.195  28.042  -0.541   0.018   0.047   0.935
B S  3BN9A                191    0.195  22.854  -1.547   0.018   0.047   0.935
B G  3BN9A                192    0.171  13.466  -2.763   0.018   0.047   0.935
B G  3BN9A                193    0.093   7.303  -0.629   0.018   0.088   0.893
B P  3BN9A                194    0.085  12.076  -1.057   0.020   0.205   0.775
B L  3BN9A                195    0.061  11.114  -0.104   0.022   0.552   0.426
B S  3BN9A                196    0.067   7.817  -0.014   0.021   0.756   0.223
B S  3BN9A                197    0.039   4.536  -0.082   0.021   0.756   0.223
E V  3BN9A                198    0.387  59.482   0.001   0.004   0.616   0.381
E E  3BN9A                199    0.367  64.115  -0.529   0.005   0.262   0.733
E A  3BN9A                200    0.582  64.191  -1.116   0.004   0.085   0.910
E D  3BN9A                201    0.722 104.040  -0.822   0.005   0.015   0.979
E G  3BN9A                202    0.380  29.922  -0.790   0.004   0.085   0.910
E R  3BN9A                203    0.307  70.395   1.832   0.004   0.514   0.481
B I  3BN9A                204    0.077  14.226   0.652   0.001   0.900   0.099
B F  3BN9A                205    0.098  19.628   1.597   0.001   0.959   0.040
B Q  3BN9A                206    0.024   4.358   0.644   0.001   0.959   0.040
B A  3BN9A                207    0.049   5.356   1.319   0.001   0.959   0.040
B G  3BN9A                208    0.028   2.204   0.018   0.011   0.918   0.071
B V  3BN9A                209    0.031   4.826   0.693   0.011   0.918   0.071
B V  3BN9A                210    0.035   5.410   0.269   0.021   0.756   0.223
B S  3BN9A                211    0.042   4.958  -0.088   0.021   0.756   0.223
B W  3BN9A                212    0.138  33.141   0.206   0.021   0.451   0.528
B G  3BN9A                213    0.101   7.941  -1.073   0.064   0.216   0.721
E D  3BN9A                214    0.357  51.487   0.585   0.052   0.084   0.864
E G  3BN9A                215    0.331  26.026  -1.216   0.113   0.087   0.800
B C  3BN9A                216    0.067   9.407  -0.368   0.053   0.043   0.903
B A  3BN9A                217    0.240  26.459  -1.022   0.058   0.017   0.925
E Q  3BN9A                218    0.452  80.745   0.760   0.018   0.019   0.964
E R  3BN9A                219    0.537 122.950   0.150   0.018   0.019   0.964
E N  3BN9A                220    0.657  96.199  -1.194   0.003   0.003   0.994
E K  3BN9A                221    0.299  61.545   0.863   0.005   0.015   0.979
B P  3BN9A                222    0.140  19.909  -0.858   0.018   0.019   0.964
B G  3BN9A                223    0.055   4.336  -1.224   0.022   0.359   0.619
B V  3BN9A                224    0.041   6.286   0.088   0.023   0.655   0.322
B Y  3BN9A                225    0.103  21.968   0.342   0.085   0.775   0.140
B T  3BN9A                226    0.042   5.812   0.442   0.087   0.683   0.230
B R  3BN9A                227    0.220  50.449   0.694   0.159   0.516   0.325
B L  3BN9A                228    0.038   6.866   0.301   0.428   0.171   0.402
B P  3BN9A                229    0.273  38.767   0.395   0.660   0.049   0.291
E L  3BN9A                230    0.356  65.202   0.923   0.717   0.014   0.269
B F  3BN9A                231    0.068  13.648   0.137   0.802   0.014   0.185
B R  3BN9A                232    0.204  46.670   1.152   0.879   0.010   0.111
E D  3BN9A                233    0.561  80.855   1.472   0.923   0.002   0.076
B W  3BN9A                234    0.123  29.461   0.754   0.970   0.001   0.030
B I  3BN9A                235    0.036   6.678  -0.528   0.970   0.001   0.030
E K  3BN9A                236    0.421  86.559   1.319   0.970   0.001   0.030
E E  3BN9A                237    0.653 114.079   1.477   0.923   0.002   0.076
E N  3BN9A                238    0.287  42.017   1.373   0.858   0.002   0.139
B T  3BN9A                239    0.206  28.544  -0.232   0.600   0.003   0.397
E G  3BN9A                240    0.714  56.223   0.282   0.115   0.016   0.868
E V  3BN9A                241    0.557  85.626  -0.931   0.003   0.003   0.994

Explain the output.  Go back.
```

---
